# Iran’s higher education and COVID-19: the “new normal” for Tehran University of Medical Sciences

**DOI:** 10.1017/cts.2021.879

**Published:** 2021-11-22

**Authors:** Amir-Hossein Memari, Ramin Kordi, Vahid Ziaee, Mohammad Hossein Pourgharib Shahi, Amir Ali Sohrabpour, Nasrin Dastjerdi, Amin Nakhostin-Ansari

**Affiliations:** 1 Sports Medicine Research Center, Neuroscience Institute, Tehran University of Medical Sciences, Tehran, Iran; 2 Division of Pediatric Rheumatology, Children’s Medical Center, Tehran University of Medical Sciences, Tehran, Iran; 3 Digestive Diseases Research Center, Shariati Hospital, Tehran University of Medical Sciences, Tehran, Iran; 4 Office of International Affairs, Tehran University of Medical Sciences, Tehran, Iran

**Keywords:** COVID-19, higher education, nerve center, pandemic

## Abstract

When the emergency committee of the World Health Organization declared that the outbreak of COVID-19 meets the criteria of a “Public Health Emergency of International Concern” on January 30th, 2020, no one could ever imagine how soon it will spread globally, and a health crisis would turn to a social crisis that affects everything including higher education. However, during this uncertainty, Tehran University of Medical Sciences (TUMS) tried to respond quickly. In this study, we explain how a nerve center has helped TUMS respond to this crisis and ensure safety to keep key operations going, and set up a functional decision-making system for the future. We also share perspectives on the critical issues, the challenges ahead, and the opportunities emerging in the “new normal.”

On March 11th, 2020, World Health Organization (WHO) declared the COVID-19 outbreak as a global pandemic [1]. This outbreak was so enormous that it turned a health crisis into an economic and social crisis [2]. On February 23rd, Iran’s Ministry of Health declared closing universities, higher education institutions, and schools in several cities and provinces [3]. However, there was a different aspect of the higher education crisis at Tehran University of Medical Sciences (TUMS) provided that our students are expected to be health-care providers and face the virus in two ways, as a person at risk of infection and as a caregiver against the infection for other people. The conditions were tough for TUMS with 11 schools, 16 teaching hospitals, and over a hundred specialized research centers committed to medical education, research, and treatment (Table 1). In these circumstances, creating an organization that provides a framework for the decision-making process to deal with the crisis was crucial.

**Table 1.** Number of Tehran University of Medical Sciences students, research centers, schools, and hospitals at the beginning of the COVID-19 pandemics

Local students	10,748 (3847 undergraduate students; 6901 postgraduate students)
International students	922 (644 undergraduate students; 278 postgraduate students)
Professors	2959 (1334 for undergraduate fields; 1625 for postgraduate fields)
Schools	11
Teaching hospitals	16
Undergraduate fields	16
Postgraduate fields	254 (124 clinical fields; 130 nonclinical fields)
Research-based fields	263

In this study, we explain TUMS’s responses to the COVID-19 crisis and how a nerve center helped TUMS respond to this crisis and ensure safety to keep key operations going and set up a functional decision-making system for the future. We also share insights on the critical issues − the challenges ahead and the opportunities emerging in the “new normal.”

## Quick Impacts of the Outbreak on Higher Education Institutions

### Health Crisis

The spread of COVID-19 has imposed special conditions on our health care systems. Engagement of TUMS students of different health professions in managing COVID-19 patients had the risk of transmitting the virus to the community besides its potential benefits [4]. In this regard, the lack of enough personal protective equipment (PPE) affected the clerkship education programs and limited the clerks’ presence in the educational wards [5].

Meanwhile, the mental health of the students should not be overlooked, as 38.1% and 27.6% of Iranian medical students suffered from mild to severe anxiety and depression, respectively, during the COVID-19 pandemic [6], which indicated a higher prevalence of these issues compared to the prepandemic situation where the global prevalences of depression and anxiety among medical students were estimated to be 20.5% and 33.8%, respectively [7,8].

### Higher Education Crisis

The specific impact of the COVID-19 pandemic on medical education was enormous since bedside learning as a major part of medical education cannot be replaced by online teaching [9]. Also, patients with emergencies rather than COVID-19 may prefer not to seek care for their diseases as they may fear contracting COVID-19 in the hospital [10,11]. There has also been a reduction in elective admissions during the COVID-19 pandemic [12], reducing learning opportunities.

### Academic Research Crisis

The implications of the COVID-19 outbreak on academic research are slightly different. Researchers across the country had to shutter their labs when universities suspended “unnecessary” research projects to follow social distancing and slow the spread of COVID-19. Instead, universities started leading research projects linked to COVID-19 and understanding its impact on different issues.

## TUMS Nerve Center for COVID-19

**Fig. 1.**
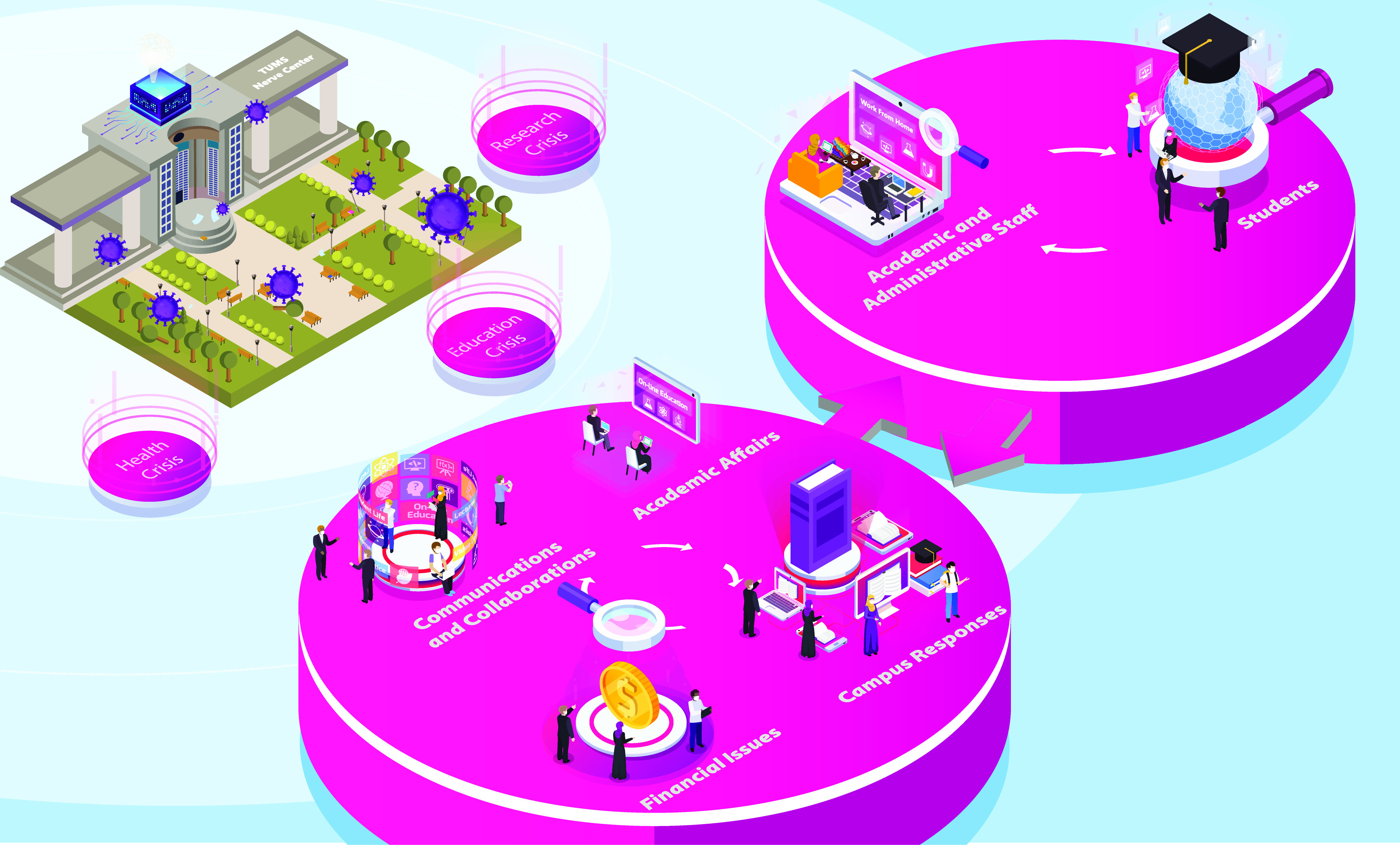
The framework of Tehran University of Medical Sciences (TUMS) nerve center in COVID-19 crisis.

After the beginning of the COVID-19 pandemic, TUMS had to provide healthcare services for people and reduce the risk of infection for its students, professors, and staff, which imposed new challenges to TUMS. TUMS needed an organization that could respond to this significant, fast-moving, and disruptive crisis. TUMS established a nerve center to provide novel solutions for the new challenges and manage the crisis in such a situation. Therefore, establishing a nerve center could help TUMS respond to the most important questions the university had in mind as it navigated the COVID-19 pandemic. Fig. 1 is adapted from the suggested framework for a COVID-19 integrated nerve center by McKinsey & Company explaining the center’s primary activities [13].

As shown in Fig. 1, the TUMS nerve center had six areas of interest. TUMS administrative staff, academic staff, and students all had representatives in the nerve center. The nerve center’s first function was to gather information related to all six domains from different TUMS schools, dormitories, and offices. On the basis of each domain’s information and challenges, the nerve center provided guidelines and coordinated these domains across the university to respond more efficiently to the crisis. Nerve center provided different solutions and suggestions for clinical and nonclinical students; however, as there was much similarity in challenges of students of different fields, these solutions were not individualized based on fields. The nerve center continuously gathered information from these domains during the pandemic to screen the functions in each domain and give feedback to related institutes and staff. Being in touch with the staff whose roles in the university were related to each domain was also necessary to recognize the newly emerging challenges during the pandemic to provide an action plan for a timely response to these challenges. The nerve center by itself had an administrative role in some cases in which it took responsibility for the management and coordination of some tasks.

### Students

#### Providing information on health and safety

Adequate, accurate knowledge is a cornerstone for appropriate action, especially in the COVID-19 pandemic critical situation [14]. Unfortunately, available information is not always accurate. About a quarter of YouTube videos on COVID-19 contained inaccurate information [15]. Also, 10% of tweets regarding COVID-19 contained false information [16]. Such studies indicate the need for a reliable source of information during the pandemic.

As a key source of accurate, science-based information, the university was to inform policymakers and the public, including students, about different aspects of the pandemic [17]. TUMS added the COVID-19 web page on the university website’s home page to provide an authentic source of the latest info on the COVID-19 virus for easy access for both the students and the public. This page, updated daily, includes guidelines on different subjects. It also refers to online resources from reputable organizations where updated information is published. TUMS took another measure to run a database to track students and news channels to provide the latest info for students, including healthcare guidelines, the disease itself, flight status, and travel restrictions. TUMS website’s traffic was evaluated daily, and the audience’s feedback on the website and social media were also evaluated and responded to as we received about 100 feedback on the website and social media daily.

#### Social media

Social media and open access to online material had critical roles in disseminating knowledge during the COVID-19 pandemic and response to this pandemic [18,19]. Considering social media as an important source of health-related knowledge and its effects on people’s healthy behaviors [20], a student-run campaign supported by international experts called “Corona Fighters International Campaign” was started to raise awareness in the region in 10 languages to combat COVID-19. The goals were defined as sharing useful lessons, experiences, management, and therapeutic solutions to overcome the crisis among health officials, health-care providers, and medical students in regional countries, countering fake news about COVID-19, stopping the spread of COVID-19 in new communities, providing evidence-based health fact sheets, technical guidelines, best practices in Iran, multimedia contents, virtual courses, online expert advice, online self-assessment, online webinar series via website and landing pages and social networks including Facebook, Twitter, and Instagram. We collaborated with our partners and graduated students overseas for maximum utilization of some social networks such as Youtube and Facebook. There were other campaigns to combat COVID-19 all around the world. For example, in the UK, medical students used Twitter to communicate and share information on COVID-19 [21], but the Corona Fighters campaign was unique in response to COVID-19 as it had a wide range of audiences from the general population to experts.

#### Support services

Inevitably, isolation, daily routine changes following the disruption to students’ lives, being far from home, and families can cause frustration, uncertainty, anxiety, and stress [6,22,23]. Using online infrastructures was one of the suggested strategies to provide mental support for students [9]. China applied a national support system to address mental issues during the COVID-19 pandemic [24]. They first used surveys to identify the populations who were at risk of psychological problems. They provided the public with online materials, including electronic books and online educational programs. Finally, they provided a long-distance counseling system on mental health issues. Together, all these steps provided a feasible approach to address psychological issues [24].

In TUMS, we ran a “Students Support System (SSS).” SSS provided different supports, including informational, emotional, educational, financial, and clinical supports. The system provides both health services via “telemedicine” and psychological services via “Tele counseling.” First and most important, medical and medications were offered to students with COVID-19 symptoms and other health conditions. Second, considering the psychological burden of the disease, emotional and social support was provided for students via “Tele counseling” psychological services.

The students who had psychological issues could use this system and benefit from a consultation with an expert psychologist. About 10% of targeted students used the provided services. We also provided informational support for students as we provided updated guidelines in several languages based on the Centers for Disease Control and Prevention and WHO advice. For international students, the latest news on visa and borders status was provided. Financial aids were offered to students who had faced financial problems during the pandemic, and we postponed tuition fee installments for some students. Also, we provided food and transfer support for students to alleviate the economic burden of the pandemic for students. Finally, online courses were held as a part of educational support. Also, a student hub was formed in SSS. In this student hub, the students supported each other emotionally, provided helpful information regarding the COVID-19 pandemic, and conducted voluntary activities and student research on COVID-19.

After imposing travel restrictions, some international students could not return to their countries and had to stay in university dorms. This group of students was at higher risk of psychological issues [23]. Misirlis et al. [25] found that loneliness has associations with depression and anxiety in international students. TUMS was in touch with embassies to ensure that international students were in an appropriate mental and physical condition. Also, the latest news on visa and borders status was provided for international students.

#### Calendar settings

After the initial postponing of the exam and classes, TUMS started offering regular courses using virtual platforms. Although this was an appropriate solution for most courses, some others could not be held in this context, such as medical clerkship courses. We scheduled online courses for those which could be held via online platforms and postponed the practical courses. The same was applicable to exams and assessments. After all, we were forced to change the academic calendar to schedule the exams and classes in a new format.

### Academic Affairs

#### Teaching policies

Many universities across the world shifted from face-to-face to online classes during the pandemic. There were several strategies to enhance electronic learning, from real-time online classes to providing and uploading educational materials [26,27]. The method of teaching in TUMS has changed too. Professors needed new skills to run virtual classes. Professors and educators needed to be prepared for such types of classes, and in some countries such as the Philippines and United Arab Emirates, courses were provided for them to prepare them for effective online delivery [26,27]. In TUMS, guidelines and training courses were provided for professors. Some courses were held using real-time classes, professors provided educational materials like voices and slides for students, and some professors were in close contact with students via online platforms to help them with their problems in the educated subjects. Dissertation defense meetings were held online to avoid gatherings. Infrastructures for online classes were also necessary to provide proper classes [26]. TUMS had held limited online courses before the COVID-19 pandemic, but the availability of these infrastructures helped TUMS immediately adopt online classes. Also, there was a virtual school in TUMS, which provided consultations, educations, and technical supports for designing and holding online courses. This virtual school supported students, professors, and managers to hold and participate in the virtual classes.

Most universities canceled or postponed their exams after the COVID-19 pandemic, but there were some exceptions. For example, in Nigeria, the National Universities Commission advised universities to fast-track the ongoing exams [26]. In TUMS, the exams were postponed after the start of the pandemic. Alternative options for evaluating students, such as ongoing evaluation during the online courses, assigning homework, and holding online exams, were considered to cope with the situation. Practical courses assessment could not be performed using such strategies and was the most challenging aspect. Reducing practical exams to the minimum possible and holding them according to health and social distancing measures was one solution to tackle this problem.

#### Introducing additional flexibilities for educational and visa affairs

As soon as the government announced lockdown restrictions, many international students submitted their exit permit requests to the university’s visa office and consulate affairs. To help with this process, TUMS held several meetings with authorities at the ministry of health, ministry of foreign affairs, and international students’ organizations to find the best solution for the new conditions considering the situation’s urgency. New rules and regulations were assigned. The staff of the office of visa and consulate affairs of the university increased work hours to process requests faster.

Adding some flexibility to educational rules and regulations was necessary to avoid pandemics’ negative impacts on students [9]. Some students had problems attending online classes due to poor internet connection or lack of facilities. Also, international students who had returned to their countries in different time zones had problems attending the real-time classes. Students were allowed to apply for academic leave in TUMS and postpone their course to the following semester.

#### Research policies

TUMS reacted to the pandemic by integrating research groups with government efforts to study epidemiological surveillance, clinical drug testing, and rapid virus detection tests. As an example, TUMS supported international collaborations and awarded grants for such collaborations. An emergency committee was established in TUMS deputy of research, and all proposals for COVID-19-related researches were evaluated in that committee. Researches, which could be conducted despite limitations related to the COVID-19 pandemic, were funded as supported, similar to the prepandemic situation. Studies that could not be conducted during the pandemic due to lack of access to subjects and laboratories’ shutdown were held or postponed.

For doing researches on COVID-19, students have had a very influential role in the whole process, from data gathering to data analysis, drafting, and publishing. Up to now, TUMS has published 118 research articles and has managed 264 research projects on COVID-related topics that most of which have been carried out with the help of students. Students’ involvement in the research projects was a learning opportunity to get familiar with the research and work under experienced professors’ supervision. Also, students’ involvement in the research projects helped academic staff manage clinical, educational, and research duties. These teams conducted several research projects on COVID-19 pandemics’ effects, for example, on industries and students’ life, helping to clarify different aspects of the pandemic [28].

### Academic and Administrative Staff

Remote working is one of the common strategies obtained by organizations, including universities, to reduce people’s contact and decrease the chance of virus transmission [26,29]. In TUMS, teachers were allowed to run their courses online from home to ensure academic and administrative staff’s health and safety. They were provided with guidelines on how to use the online platform for their lectures. Administrative staff at risk were allowed to work from home, and others were allowed to work for fewer hours.

### Campus Responses

Like many other universities worldwide [26], TUMS postponed or canceled all campus events such as workshops, conferences, sports, graduation, and other activities to avoid mass gatherings and take intensive measures to protect all students and staff members.

We had to keep some dormitories open since about 450 of our international students could not go back home because of travel restrictions. First, the hostel buildings were sanitized and, all students were screened for COVID-19 via physical exams. Guidelines on personal healthcare, social distancing, self-isolation, exit, and entry were prepared. Sanitizers, masks, gloves, and other required materials were provided. The hostel staff was trained. All to make sure that students are safe and secure. Food was served at the hostel every day to reduce daily shopping.

Providing appropriate technology for hostels and campuses was an important issue. Students needed a high-speed internet connection to attend online classes. They also needed high-quality webcams to make use of telemedicine.

### Financial Issues

As one of the main sources of income for educational universities in Iran, hospitals faced economic crises during the pandemic. Reduction in the number of outpatient visits and elective surgeries and additional resources to prepare the staff and hospital to visit and admit COVID-19 patients lead to hospitals’ financial pressure [30]. Iranian universities also had some limitations regarding transferring money to and from the banks abroad due to sanctions, which could also affect international students’ payments. Although TUMS was experiencing financial constraints, we considered flexibilities on the financial status of students. For some students who had economic problems, a part of the tuition fee was waived to help them continue their education during this crisis. Despite being in debt, some others were allowed to leave the country to be at home with their families. We also agreed to postpone tuition fee installments for some students. For the students who could not leave the country and stayed at university hostels, TUMS helped them with living expenses. Also, in collaboration with some embassies, the evacuation of students was supported by the university.

### Communications and Collaborations

Establishing a specific webpage for information on COVID-19 helped expand connections to the local community and the international one. Providing authentic information in several languages, running online webinars with international experts, publishing the latest researches, documenting activities, producing multimedia content about best practices, and experts’ experiences were all measures that were in place to keep the connection with the world.

One of the special functions in this era was volunteers who were played by academics, students, alumni, researchers, and local communities. In England, medical students helped the health care system in the medical and nonmedical settings as they helped distribute PPEs and other activities to control the outbreak [31]. From the first days of the COVID-19 outbreak in Iran, TUMS hospitals offer health and treatment services to people. TUMS alumni have played a significant role by offering endowments to hospitals and health centers and participating in webinars and scientific activities.

## New Normal Rather than Returning to Normal

It is not the first time higher education faces a crisis, but this one seems challenging. As it is with any other crisis, there are challenges and opportunities, hopes, and uncertainties. This pandemic is adding a higher degree of complexity to higher education.

### Financial Uncertainty

One of the immediate impacts of this outbreak on higher education institutions has been financial uncertainty. The decline in the number of international students who were forced to quit their courses due to financial crisis and travel restrictions is an issue that is more prominent in developed countries with more advanced education systems as they recruited a significant number of students from other countries annually [32]. Also, shifting to online education systems and online courses makes the situation more complicated for universities with no adequate electronic learning [32]. As one of the main sources of income for medical sciences universities, hospitals also face a financial crisis, leading to further economic pressure on universities [30]. For sure, universities will need to work hard on financial sustainability. Governmental financial supports during the COVID-19 pandemic can be a short-term solution for universities [33]. Also, universities should plan for new income sources, such as virtual courses, suitable for pandemic and postpandemic situations. Using available free online platforms, such as Zoom and Google Meet, also helped TUMS saving financial resources.

### Distant Teaching

Shifting to online teaching was a quick response for universities right after campus closures. While it was a short-term decision to keep education continuity and university-students communication, it can cause secondary problems in the long term. Also, not all universities can run an online learning system.

Now traditional, campus-based universities should choose the right technologies to improve their digital infrastructure. This change is followed by further investment while funding is tight. Even if appropriate infrastructure is provided, both the academic staff and students’ technical adaptability is questionable.

Furthermore, online teaching can challenge sustaining the values of higher education, such as equity and equality. Students are back to their homes that are not fully equipped with technology and infrastructure. Therefore, students who have access to insufficient resources for communication technology will be left behind.

### International Collaboration

Apart from travel bans and locking borders, global collaborations among researchers around the world have been shaped. Now it is time for tertiary education institutions to act autonomously and focus on their primary responsibility, producing sciences. The world needs their help to fight the pandemic and survive. By working together, we can formulate good policies to benefit people worldwide and come together across boundaries.

### Volunteer Communities

While governments, the health sector, and industry are working hard to respond to this crisis, social mobilizers, community workers, and volunteers have an essential role in providing help. In TUMS, medical students, interns, and residents joined the health workers team for a common purpose. Medical students voluntarily helped in research projects, providing and distributing the PPE, providing educational content and guidelines, and raising awareness in the community. Medical students’ involvement in medical and non-medical settings in the UK to combat COVID-19 crisis [31] and the student-run campaign in TUMS to raise awareness indicate that everyone has valuable and vital roles in their positions, which can help for better management of such a crisis.

## Refining Traditional Model of Education

This crisis can be a good opportunity for a fundamental reexamination of higher education. In this process, higher education should build resilience against crisis downturns to ensure sustainability. The ability to communicate readily and frequently with the university community and provide a safety net for its most vulnerable students is of great importance. Therefore, providing a strong support system for both students and academics who are health workers is crucial. Considering TUMS as an example, in the COVID-19 pandemic, higher education institutions have shown their significant capacity to adapt very quickly to the crisis. However, how many of them had anticipated this type of crisis, had put sophisticated risk management in place, and had worked on scenario planning for it? Education was on the road to change and evolve to the next level before the pandemic, but now the pace of transition is much higher, and our institutions should be ready for massive changes or even a historical metamorphosis.

## Conclusion

Like all universities, TUMS needed to keep pace with these accelerated changes due to the COVID-19 pandemic and prepare itself for the “new normal,” Nerve center helped TUMS in two aspects; first, it helped the university to manage the crisis. Second, it helped to university to learn how to continue its activities with the new normal. During the pandemic, TUMS tried to provide a proper context for students’ effective education in the rapidly changing situation as the university’s primary function. Also, students’ involvement in the campaigns to combat the pandemic, their active role in the decision-making processes, and their role in the clinical management of COVID-19 patients, was a valuable opportunity to become familiar with their future roles in such crises.
